# Consumers as tutors – legitimate teachers?

**DOI:** 10.1186/1472-6920-4-14

**Published:** 2004-09-20

**Authors:** Cathy Owen, Rebecca E Reay

**Affiliations:** 1 Medical Education Unit, Medical School Australian National University, Australian Capital Territory, Australia; 2 Frank Fenner Blg 42, Canberra ACT 0200 Australia; 3 Academic Unit of Psychological Medicine, Blg 15, Level 2 The Canberra Hospital, WODEN ACT 2605 Australia

## Abstract

**Background:**

The aim of this study was to research the feasibility of training mental health consumers as tutors for 4^th ^year medical students in psychiatry.

**Methods:**

A partnership between a consumer network and an academic unit in Psychological Medicine was formed to jointly develop a training package for consumer tutors and a curriculum in interviewing skills for medical students. Student attitudes to mental health consumers were measured pre and post the program. All tutorial evaluation data was analysed using univariate statistics. Both tutors and students evaluated the teaching program using a 4 point rating scale. The mean scores for teaching and content for both students and tutors were compared using an independent samples t-test.

**Results:**

Consumer tutors were successfully trained and accredited as tutors and able to sustain delivery of tutorials over a 4 year period. The study found that whilst the medical students started with positive attitudes towards consumers prior to the program, there was a general trend towards improved attitude across all measures. Other outcomes for tutors and students (both positive and negative) are described.

**Conclusions:**

Consumer tutors along with professional tutors have a place in the education of medical students, are an untapped resource and deliver largely positive outcomes for students and themselves. Further possible developments are described.

## Background

Interview skills, while important in all areas of medicine, are a prime focus of educational endeavour in the teaching of psychiatry. The interview is the cornerstone of psychiatric investigation and the scene for the establishment of rapport and therapeutic engagement. The importance of effective skills training in interviewing for medical students was highlighted in the core curriculum in psychiatry published by The World Psychiatric Association and the World Federation for Medical Education [1]. An additional perspective on this issue has come from consumers who report a distinct difference between effective and ineffective interviewing styles, highlighting the hindering effect of bored, impersonal interviewers who make judgemental assumptions about an individual's behaviour [2].

Moving beyond the traditional teaching of interview skills by psychiatrists in the "see one, do one, teach one" mode, we report the development of an innovative approach to the teaching of psychiatric interview skills. This approach began four years ago as a partnership between mental health consumers and academic psychiatry to examine the ongoing feasibility of training mental health consumers as tutors for 4^th ^year medical students in psychiatry. The idea is based on the belief that consumers have a legitimate experience to share and a rich skill base on which to draw. The purpose of this ongoing project is to contribute to the ultimate development of a workforce of medical practitioners with clinical assessment skills that are better tailored to the needs of mental health consumers. More immediate potential benefits are the promotion of an engaging curriculum in psychiatry for students, offering direct meaningful contact with mental health consumers.

### Literature review

Consumers and patients are not new to medical education, offering a unique perspective on their experiences with the health system. There are 3 levels of consumer participation in education. Consumers may be the subject of teaching tutorials (a demonstration), a visitor to a tutorial (sharing their experiences with few guidelines) or a paid trained tutor delivering an agreed curriculum (a 'consumer tutor'). Consumer tutors are distinct from 'professional tutors' (those with a career obligation to teach) a distinction that does not imply a lack of professionalism regarding the consumer tutor. The issue of language is not trivial: the local user support network was committed to the term 'consumer' rather than other common labels like client or patient. The language used was chosen for the perception of action and autonomy and to reflect respect for the role and to make it clear to students and staff that these people were a committed part of the teaching workforce, not visitors only present to tell of their (often negative) experiences.

This work drew on the Partners in Arthritis project [3] that demonstrated that arthritis patients are at least equal to Consultant Rheumatologists in the teaching of examination techniques for arthritis. Related work included the use of families with experience of paediatric illness in the growth of communication skills for medical students [Reynolds 2003: personal communication] and the use of clinical teaching assistants in pap smear training [Vivienne O'Brien 2003: Personal communication]. Consumer and carer involvement in mental health education has been documented: nurse education by a consumer academic [4] with largely positive outcomes, user and carer involvement in curriculum development and delivery in interprofessional postgraduate mental health education [5], a randomised trial of brief mental health staff training by consumers with positive post-training attitudes from those taught by consumers [6] and a reminder that there are few published examples of the translation of policy rhetoric regarding consumer and carer involvement in education into teaching reality [7]. In the local area, consumers are involved in Mental Illness Education ACT (MIEACT), a national non-profit outreach education project to high school students and community groups, which aims to reduce stigma and improve mental health literacy of young people [8]. In all these projects consumers were trained, assisted in ongoing curriculum review and contributed their lived experience in the domain of interest. Despite this fertile environment for the establishment of this project and the growing consumer and carer action in psychiatry in general, there was no literature identified using trained consumers in teaching psychiatry to medical students.

In summary this project set out to train and support mental health consumers as consumer tutors for the delivery of a jointly developed curriculum for 4^th ^year medical students in effective approaches to interviewing. There was commitment to evaluate the effectiveness of the teaching in terms of (i) changing student attitudes towards consumers, (ii) preparing students for a common examination in psychiatry, (iii) monitoring consumer tutor involvement and feedback.

### The Setting

The project was set in a medical school (University of Sydney) undergoing transition to graduate entry, with adult self-directed and problem based learning models providing an opportunity to students keen to explore new ways of learning. This environment of educational change added to student and staff enthusiasm to trial new ideas. There was recognition that carers and consumers required the same respect and courtesy as professional tutors, and inequities such as only being paid travel expenses were unsatisfactory [9]. Consumer consultant positions were established to improve the relevance and user friendliness of psychiatry services using trained, paid and supervised staff [10].

## Methods

### Phase 1: Curriculum development

The Academic Unit of Psychological Medicine approached consumers from the local mental health consumer network seeking expressions of interest to join a steering committee to oversee the project. Consumers were involved at all stages including planning, development, implementation and evaluation. The steering committee met weekly to determine an approach to consumer tutor recruitment and training, and to author the student curriculum for delivery.

### Consumer tutor development

Consumer tutors were recruited, trained in small group methods and assessed. The assessment task was a nine-item quiz. Typical questions included "List three strengths of working in small groups" and "List three strategies to get a discussion going amongst students". Those who wished to teach were then allocated in pairs to a consistent student group of six to eight students for weekly tutorials over seven weeks. Consumer tutors conducted the planned curriculum, provided feedback on each tutorial and contributed to curriculum review.

Evaluation of the content of the curriculum, the quality of the teaching and handouts was measured using a 4-point Likert scale (4 = excellent, 3 = good, 2 = fair, 1 = poor) followed by an open comment item. After each tutorial consumer tutors were debriefed by academic staff and arrangements for the following tutorials confirmed. An expedient payment method was established. Over time, consumer tutors and academic staff periodically reviewed tutor and student feedback and decided on tutorial modification.

### Partnership establishment

The steering committee comprised six consumers, three with previous teaching experience at secondary or tertiary level. Professional approaches were adopted for recruitment, training, assessment, graduation and payment. Academic staff drafted a structure for consumer tutor training completed by the committee. The committee determined the priorities for the student curriculum and each oversaw the writing of a tutorial in conjunction with academic staff. Consumer input was central and meaningful rather than at the level of editing academic staff work. The committee were paid regular sitting rates for committee attendance. The steering committee met during the first trial to review progress, an overseeing role replaced later by the consumer tutors themselves.

### Consumer tutor recruitment and training

Expressions of interest from potential consumer tutors were sought through the consumer network against selection criteria. These included the essential criteria of being a current consumer of mental health services and having an interest in the development of medical student interviewing skills. Desirable criteria included previous experience in teaching. Applications were reviewed by the steering committee and all applications for training were accepted. Payment for training and tutoring was set at the current university casual tutor rates appropriate to qualifications.

Recruitment resulted in a cross section of consumers including several 'marginalised consumers' who are usually under represented in such activities Three training cycles have occurred with 20 consumers (12 women, 8 men) commencing training and 18 consumers graduating from training. Fifteen consumer tutors have taught over the four years, whilst three decided after graduation that they did not wish to teach. Drop out occurred because of illness, disinterest or realising that tutoring was more difficult than first imagined.

The tutor training program comprised six weekly, one and a half hour tutorials delivered by academic staff encouraging discussion, controversy and practice in a supportive environment. See Table 1 for a summary of the consumer tutor training program. A graduation ceremony was conducted and the Dean of the clinical school awarded university badged completion certificates, although the university did not formally accredit the course. Trainee tutor evaluations revealed they valued the training experience, reporting a sense of initial nervousness later replaced by a sense of assurance of their own abilities.

**Table 1:** Content of 'consumers tutor' training program

**Session**	**Topic**	**Content**
1	Orientation to 4^th ^year medical students	What have they learnt so far? What other teaching and experiences do they receive during the psychiatry term? What are the characteristics of medical students?
2	Working in small groups	Why are groups effective learning settings? How do groups work? How do you manage dominant and quiet group members?
3	Giving effective feedback	Strategies to improve giving and receiving feedback with hands on practice
4	Review of the student curriculum	Practice delivery of the planned student material through small group role-play
5	Review of the student curriculum	Practice delivery of the planned student material through small group role-play
6	Trouble shooting, problem solving and tutor assessment	Common fears amongst the trainee tutors? What will go wrong? How to deal with inability to attend? Completing the written assessment

### Phase 2: Curriculum delivery

#### Student participation

Students in one of four teaching centres of the University of Sydney participated in the consumer tutor-led tutorials. Students were oriented by academic staff and completed a pre-participation measure. This measured student attitudes to mental health consumers adapted from work on the 'hated patient' [11]. Five statements were rated on a five point Likert scale (strongly agree to strongly disagree). Typical items included "I value learning from consumers" and "I would like mental health consumers as part of my practice." The final item was an open-ended question about concerns in interviewing.

Students were then introduced to their consumer tutors and participated in an 'ice breaker' session involving an interactive board game designed to sensitise medical students to the experience of being a mental health consumer. This was followed by six tutorials which both consumer tutors and medical students evaluated using the same measure. Students repeated the attitude measure at the end of the program and completed assessment tasks as per the university requirements. Students also participated in a seminar series (including didactic teaching on interview content) with professional tutors similar to that delivered in the other teaching centres. All tutorial evaluation data was analysed using univariate statistics. The pre and post attitude measure was compared using term group means (as completion was anonymous) using an independent samples t-test. The mean scores for teaching and content for both students and tutors were compared using an independent samples t-test.

## Results

### Delivery of the student curriculum

The student curriculum was developed as six one-hour tutorials (see Table 2 for a summary).

**Table 2:** Content of Consumer tutor-led tutorials for medical students.

**Session**	**Title of session**	**Content**
"Ice breaker"	Lemon Looning Board Game	An interactive game designed to simulate the experience of a mental health consumer.
1	'Person centred interviewing'	Discussion about medical student concerns re interviewing, dealing with fears and previous experience of psychiatric contact. Role play- Practicing sensitive interviewing styles
2	'Dealing with sensitive issues'	Further developing effective interviewing styles for the purpose of exploring social and family circumstances, talking about lifestyle including sexuality, drug use, relationships and parenting.
3	"Reality Check"	Dealing with fixed beliefs and delusions. Using the therapeutic relationship to enhance understanding of patients affected by delusions and hallucinations.
4	"Art Express"	Developing skills in talking about self harm. Activity: using an art therapy exercise to more effectively respond to people who are depressed.
5	"Bringing it all together"	Practice interviews with volunteer in-patients and using peer discussion for feedback. Aims: practicing sensitive interview skills for history taking.
6	"Dealing with the unexpected"	Practice interviews with volunteer in-patients and using peer discussion for feedback. Aims: dealing with time constraints, dealing with challenging or unexpected behaviour, effectively closing an interview

The tutorials were delivered by pairs of consumer-tutors to small groups of six to eight medical students. The tutorials were graded in terms of level of difficulty beginning with general discussion of sensitive interviewing styles, role playing with tutors, followed by live interviews with volunteer inpatients from the psychiatric unit. Each tutorial included discussion of the pertinent issues, practice, review by the practicing student and feedback from peers and consumer tutors. The consumer tutors independently facilitated and participated in the tutorial without the involvement of academic staff who was available to assist if needed. They were rarely required (usually to resolve room double bookings). The curriculum and written materials underwent several revisions in response to feedback aimed at improving interactivity and clarity. Consumer tutors used the ground rules set out in training for dealing with absences. Reserve tutors were introduced at the start of the term so they were familiar to students if required. Tutor pairs were able to support each other and compensate for ebbs and flows in performance.

The consumer tutors debriefed following each tutorial with academic staff. These meetings were an important opportunity for tutors to give positive and constructive feedback to each other as well as addressing ways to improve their delivery of the tutorials. They discussed problems and obstacles and brainstormed effective solutions. The larger consumer tutor group contributed to successfully resolving most conflict. Discussions were frank. Formal mediation was used to resolve a conflict between two tutors, in dispute over matters beyond teaching. Mediation was successful in terms of allowing ongoing involvement in teaching for both people. Academic staff informally debriefed the students during other tutorials. While a few students complained about the whole experience of being taught by a consumer, most students were positive. Indeed many reported they used the consumer tutors as a sounding board for other interview-related experiences during the week, seeking advice about alternative styles and approaches.

### Tutor maintenance

Like all tutors, the consumer tutors required support, stimulation and refreshment. This happened in the tutorial debriefs outlined and in occasional workshops to review feedback, revise curriculum and refresh skills. Consumer tutors were encouraged to present reports of their experiences at appropriate meetings and received a national award for consumer research. Consumer tutors' motivation particularly increased when clinical mental health staff asked them about their teaching experiences and recognised that the individual had made many gains since the last episode of acute care.

Consumer and professional tutors were commonly concerned about intervening illness and the impact on teaching. Some consumer tutors became acutely unwell during the term and required care. As a result they developed an agreement to postpone their involvement in the tutoring whilst they received necessary treatment. Consumer tutors and students were understanding of this and students had a rare experience of the longitudinal patterns of an illness and the person.

### Student attitudes

Out of a total cohort of 104 medical students, students completed the pre (n = 72) and post (n = 68) attitudes questionnaire using a 5 point scale (5 = strongly agree, 4 = agree, 3 = uncertain, 2 = disagree, 1 = strongly agree). A comparison of mean scores on 3 items reflecting student attitudes towards consumers was conducted using an independent samples t-test. Results showed that prior to the program the medical students began with positive attitudes towards learning from consumers (n = 57, x = 3.89, s.d. = .865) and working with mental health clients (n = 72, x = 3.68, s.d. = 747). Whilst there was a general trend towards further improvement in their attitudes, their mean scores pre and post the program were not significantly different. However, the medical students did show a significant improvement in their belief that "clients in psychiatric units give reliable histories" (n = 72, x = 3.07, s.d. = .657, p < .005) (see Table 3). This general improvement in attitudes to learning from and working with consumers was reflected in the open comments (see sample of comments in Table 4).

**Table 3:** Comparison of mean scores of student attitudes pre and post the program.

**Statement**	**Pre and Post**	**N=**	**Mean**	**Std. Deviation**	**Sig. (2-tailed t-test)**
"I value learning from mental health clients"	Pre	72	3.89	.865	.731
	Post	68	3.94	.929	
"Clients in psychiatric units give reliable histories"	Pre	72	3.07	.657	.005
	Post	66	3.42	.805	
"As a doctor I would like mental health clients as part of my continuity of care practice"	Pre	72	3.68	.747	.084
	Post	68	3.91	.824	
Total	Pre	72	10.64	1.550	.063
	Post	68	11.18	1.836	

**Table 4:** Sample of medical students' open comments pre and post program

**Pre training**	**Post training**
"For our purposes, consumers lack the ability to instruct us with relevant information."	"The consumers give us insight into what it is like to be on the other side of the mental health system. This is invaluable in helping us to be better doctors and increase our empathy."

In addition, students' greatest concern regarding interviewing mental health consumers changed before and after the teaching. Before, students were preoccupied with violence in the interview. At the conclusion of the program, students remained concerned about violence and unpredictable reactions. However, they reported increased concern with their ability to build rapport, engage and understand the client. The program has since been modified to address their concerns about violence early on in the training.

### Tutorial evaluations

Tutor and medical student evaluations using a 4-point Likert scale (4=excellent, 3=good, 2=fair, 1=poor) were returned on 452 occasions. Analysis of the mean scores of students on the quality of the 'teaching' and 'content' of the program revealed favourable ratings ('teaching' n = 450, x = 2.81, sd.76; 'content': n = 451, x = 2.82, sd= .689). Whereas, tutors tended to rate the program even higher ('teaching': n = 372, x = 3.08, sd = .510; 'content': n = 369, x = 3.14, sd=.496). A comparison of the mean scores of students and tutors using an independent samples t-test showed that this difference was statistically significant (p < 0.001), see Table 5. Open comments about the program varied as shown in Table 6.

**Table 5:** Analysis of mean scores on the quality of the 'consumers as tutors' program.

	**Tutor/student**	**N=**	**Mean**	**Std. Deviation**	**Sig. (2-tailed t-test)**
Content	Student	451	2.82	.689	.000
	Tutor	369	3.14	.496	
Teaching	Student	450	2.81	.760	.000
	Tutor	372	3.08	.510	

**Table 6:** Open comments about the quality of teaching:

I am impressed with the astute feedback which is encouraging and critical"- Medical student.
"It was helpful. The tutors explained that even if the interview was going nowhere to keep persisting gently as the patient is just sizing you up"- Medical Student.
"The consumers are a valuable source of encouragement and feedback"- Medical student.
"The students interviewed a patient and showed warmth, good questions and rapport. He did not press when the patient did not want to divulge"- Consumer- tutor.

### Assessment

All medical students who participated in the consumers as tutors program passed the university wide assessment of an observed psychiatric interview rated against defined criteria.

## Discussion

This project established the feasibility of training and supporting mental health consumers as tutors for delivery of a jointly developed curriculum for 4^th ^year medical students in effective approaches to interviewing. Training and delivery has continued requiring modest maintenance, perhaps in keeping with sustaining professional tutors. Consumer tutors have shown themselves to be reliable, professional in approach and amenable to feedback. Benefits for students (as measured in their open evaluations) included the extended experience of working with a consumer of health services, the development of a clearer perspective regarding consumer views and an opportunity to see people with mental illness in recovery. Students were at least as well prepared as their peers for a structured assessment in interviewing (from the combined effect of traditional and novel teaching). Students largely reported positive experiences, found the curriculum and delivery acceptable and saw tutor experience and knowledge as legitimate and valuable. Ideally it would have been useful to follow up medical students over a longer term to assess their psychiatric interviewing skill, however, this was not practically possible within this study.

The attempt to measure attitudes deserves discussion. Attitudes are recognised as an important component of curriculum development yet remain the personal business of each of us. It would be reasonable to see education as a means of working past one's own attitudes rather than seeking to refine or replace student attitudes. Guidelines for working with consumers in health care assume that "for consumer participation to be effective, all participants in the process need to respect the different skills and expertise of the other participants" [12]. In this study, student attitudes to consumers had a tendency to improve across all dimensions measured. On average the medical students began the program with largely positive attitudes to working with and learning from consumers which may explain the lack of statistically significant difference in their attitudes pre and post the program. In addition, a finding of lack of significance using a pre and post test design with a small sample of subjects is not usual. The one attitude measured that did improve throughout the program and reached statistical significance was towards mental health clients in psychiatric wards. This finding is understandable in light of the fact that the training took place within a psychiatric unit and the program incorporated practice at live interviews with clients from the unit. In addition, the study found a change in the primary focus of medical student concerns regarding interviewing which moved from issues focused on the consumer (such as violence or unpredictability) to those focused on improving their skills in interviewing and seeing this as a worthwhile activity. In terms of their satisfaction with the training program, based on their open comments, the few students who objected at least had the challenge of working in an educational model they did not admire. This was thought provoking and engaging even if the response produced was negative.

Benefits for consumer tutors (as measured by their open evaluations) included enhanced self esteem and financial reward for work done. Consumer tutor curriculum development was novel such as utilising an art therapy vehicle to experience a non-pharmaceutical therapeutic device. Most consumer tutors have continued to teach, with appropriate breaks, and have mentored new tutors. Some have used this experience to step further into paid employment and to rehabilitate previous work skills. Consumer tutors have remained resilient and episodes of relapse appear to be multifactorial in origin (with teaching perhaps one of the factors). This robustness was also found in a study of psychological impact on consumers working in a peer support role in an acute care setting [13]. Consumer presentations have centred on the powerful personal effects of participation in learning new skills and gaining confidence. The largely positive ratings of tutors about the program was not as positive as the medical students, highlighting the need to evaluate both groups to adequately measure the effectiveness of the program.

Benefits for the health system included the placement of consumers in a 'professional' light. Consumer tutors shared the staff tearoom, were paid as other casual tutors and were seen as well contributors rather than being in the sick role. Professional tutors were aware of the consumer tutor teaching and perhaps viewed it as 'politically correct' rather than educationally effective. Dissemination of findings via service and conference presentations has helped address this common view.

Tutoring medical students is a skilful and potentially stressful role, and is not suitable for all mental health consumers. Following the training program some trained tutors realised that teaching was not their interest or strength (a proportion of whom did not teach at all). This was anticipated and should be factored into training plans. Some consumer tutors realised their tolerance level was insufficient to manage student junior skill level and found it hard to resist retelling their 'war stories' of difficult clinical encounters. This was a common theme in debriefing and required active refocusing on the curriculum of effective and ineffective interview techniques. The occasional protesting student required gentle persuasion to see that ongoing participation was a way of exploring contact with consumers. Like most education programs this approach did not run itself. Tutors required sustenance; feedback needed action and materials needed review. New tutors needed to be trained to add to the growing pool of available people. Despite these issues, our experience was that this was manageable and in keeping with maintenance of quality teaching by professional tutors.

We believe this model is another valuable option in a range of consumer involvement programs and could be replicated in health, emergency services and support agency education. Discussions have occurred with carers about possible involvement. At this stage it was decided to invite carers to speak about specific topics in the mainstream program as consumers see their expertise as fundamentally different to that of a carer. Despite the potential for use with other groups, our attempt to use this experience in refreshing interview skills in general practitioners was unsuccessful. Notwithstanding the diligent work by all parties, local general practitioners held fast to the view that consumer tutors would lack the emotional robustness to survive teaching. They were welcome to come and talk of their experience but were not seen as competent to deliver a curriculum. This may well have been shorthand for more complex issues of concerns about confidentiality, power and autonomy. This example reminds us, however, that health education is in change and that new strategies are required to engage today's students in experiences that will produce clinicians skilled to support effective consumer participation in healthcare.

## Conclusions

We have detailed a feasibility study which demonstrates a new level of consumer participation in the design, implementation and evaluation of a medical student training program. The effort has been sustained over four years with appropriate maintenance. Largely positive outcomes were seen for students, consumer tutors and the health care system. These included raising the profile of consumers as 'legitimate teachers' in medical education and contributing to an improvement in the attitude of medical students towards mental health consumers. Together the joint partners in the program were able to manage obstacles, such as, pessimistic attitudes towards the involvement of consumers and difficulties adhering to the curriculum. Adopting a continuous review of the feedback from both medical students and consumer tutors has helped to further refine our ability to deliver the curriculum and better support the participants. Lastly, our experience has been that consumer tutors are an untapped resource offering a richness of experience and a professional approach to teaching that deserves closer examination in other health settings.

## Competing interests

Pfizer funded the initial pilot in the first year of the study.

## Authors' contributions

CO conceived of the study, participated in the design of both the consumer tutor curriculum and medical student curriculum and performed the data analysis. CO and RR participated in the training of consumers, monitored the implementation and evaluation phases and coordinated the program. Both authors read and approved the final manuscript.

## Pre-publication history

The pre-publication history for this paper can be accessed here:


